# Medical News and Bulletin

**Published:** 1843-04-29

**Authors:** 


### MEDICAL NEWS AND BULLETIN.

We shall commence at an early date the 'Clinical Lectures on the Diseases of the Genital Organs of the Female,' now delivering at the Hôpital de Lourcine at Paris, by Dr. Huguier. This course was commenced on the 20th of February last, by special permission from the Hospital Administration. The number of tickets issued was limited to twenty-five, and the class was composed almost entirely of graduates, who showed a great desire to repair a want that they had experienced in their studies, and thus complete their medical education.

---

We have received the "Announcement of the Private Lectures of Heber Chase, M. D., with a Catalogue of the Classes from their commencement in 1838."

---

A little work has just been published in this city with the following title: "The Physician's Pocket Dose Book, and Student's Manual, comprising all the important articles of the Materia Medica, with their appropriate doses. By Thomas T. Smiley, M. D." It professes to be based on the classification of Professor Wood.

---

In the Annual Announcement of the Medical Department of Transylvania University for 1842–'43, we observe that the number of students in the Institution for the past session was two hundred and four, and the number of graduates sixty.

---

### THE NEW YORK JOURNAL OF MEDICINE.

This is the title of a new journal to be commenced on the 1st of July in New York, and to be edited by Samuel Forry, M. D., already well known to the profession by his work on "The Climate of the United States." It is to be published every alternate month, and each number will contain 144 'ample' octavo pages. Subscription, $5 per annum, in advance.

As the organ of the profession in New York, and the repository of their valuable contributions to our science, we trust that it will meet with entire success.

The Messrs. Langley's, 57 Chatham street, are the Publishers.

---

### PHILADELPHIA HOSPITAL.

At a meeting of the Board of Guardians, &c., held on the 17th inst., Meredith Clymer, M. D. was elected an Attending Physician to the Philadelphia Hospital, Blockley, in the place of C. W. Pennock, M. D., resigned.

---

### M. BOUILLAUD.

M. Bouillaud has been elected, for the third time, member of the Chamber of Deputies for Angouleme. The two previous elections had been declared void through informality.

---

### DR. HOPE AND M. LIEBIG.

The "Gateshead Observer" mentions a report that Dr. Hope will retire from the chair of chemistry in the University of Edinburgh at the close of the present session, and be succeeded by Professor Liebig.

### STRUCTURE OF THE UTERUS.

M. Jobert endeavours to show that the uterus is formed by a *single* muscle, the fibres of which take various directions.

---

### DEATH OF DR. MACARTNEY.

We have to announce, with feelings of the deepest regret and sorrow, the death of Dr. Macartney, of Dublin, which took place on Sunday last, at his residence in Merrion-street, Dublin. The cause of this melancholy event was probably apoplexy, for Dr. Macartney was left in a state of excellent health on Saturday night, and was found unconscious on the following morning.

---

### ANECDOTES OF BERZELIUS.

When Berzelius returned he received us with great cordiality. We were much struck with his appearance—judging by the appearance of some of the German *savans*, we had expected to find him an odd out-of-the-way kind of being; but he is totally devoid of affectation either in dress or manner. Men of eminence in Germany startle the stranger in quite a different way; they may look like men of genius, but they would seldom be taken for men of sense. Their pale faces, long "unkempt" locks, and antiquated garments, afford the most complete contrast to the healthy looks and unaffected bearing of this Swedish rival. In fact, from his dress, ease of manner, and total want of pretension, he might pass in any society in Europe, not for Berzelius the great chemist, but Berzelius the well-bred gentleman. In place of Dr. Faustus' garments, he sports a smart carriage cap, silk vest, and blue coat, very like those of ordinary mortals. He is a well made good-looking man, of the middle size, rather stout than otherwise, but with nothing in his appearance to make us suspect that he had gout, and found it necessary to drink chalybeate water. In a visit to Paris, the preceding summer, they had tried to kill him with kindness, but judging by his looks, we should say he will survive many such assaults; he travels much, and proposed an early visit to Copenhagen.

If his manner be unaffected, his conversation is equally so: it has nothing of *the shop* about it. Not that he shunned—for that in him would have been affectation of the worst kind—all allusion to his own science. Part of the conversation (which was carried on chiefly in English with the aid of an occasional theft from German) turned on *our* eminent scientific men; and nothing could have been more becoming than the liberality with which he praised these, his worthy fellow-labourers. Faraday, Buckland, Sedgwick, Jameson, all came in for the deserved meed of approbation; but the Wernerians of course did not escape without a gentle pat. When he remarked that their warm attachment to the principles of their school was in a great measure attributable to their affection to its head, whom his pupils worshipped as a kind of deity, and, therefore, regarded every departure from his lessons as sacrilege, it struck us that the same may soon be said of himself and of his school. His pupils revere him with boundless affection; but they must expect in their turn to be termed "antiquated."—*Bremner's Excursions in Denmark, Norway, and Sweden.*

